# Cognitive and Linguistic Benefits of Aerobic Exercise: A State-of-the-Art Systematic Review of the Stroke Literature

**DOI:** 10.3389/fresc.2021.785312

**Published:** 2021-12-24

**Authors:** Jamie F. Mayer, Chaleece W. Sandberg, Jennifer Mozeiko, Elizabeth B. Madden, Laura L. Murray

**Affiliations:** ^1^School of Allied Health and Communicative Disorders, Northern Illinois University, DeKalb, IL, United States; ^2^Department of Communication Sciences and Disorders, Penn State University, University Park, PA, United States; ^3^Department of Speech, Language and Hearing Science, University of Connecticut, Mansfield, CT, United States; ^4^School of Communication Science and Disorders, Florida State University, Tallahassee, FL, United States; ^5^School of Communication Sciences and Disorders, Western University, London, ON, Canada

**Keywords:** stroke, aphasia, aerobic exercise, cognition, language, rehabilitation

## Abstract

This systematic review aimed to determine how aerobic exercise affects cognition after stroke, with particular focus on aphasia and language improvement. Methodological quality was assessed with the PEDro+ scale with half of the 27 included studies rated as high quality. Data extraction focused on cognitive effects of aerobic exercise post-stroke, intervention characteristics, outcome measures, and participant characteristics. Whereas attention, memory, and executive functioning measures were common across the included studies, no study included a language-specific, performance-based measure. Seventeen studies reported positive cognitive effects, most frequently in the domains of attention, memory and executive functioning. Variability in outcome measures, intervention characteristics, and participant characteristics made it difficult to identify similarities among studies reporting positive cognitive effects of exercise or among those studies reporting null outcomes. Only three studies provided specific information about the number of individuals with aphasia included or excluded, who comprise approximately one-third of the stroke population. The review identified patent gaps in our understanding of how aerobic exercise may affect not only the cognitive domain of language post-stroke but also the broader cognitive functioning of individuals with post-stroke aphasia. Methodological limitations of the reviewed studies also warrant further examination of the direct impact of aerobic exercise on cognition post-stroke with careful attention to the selection and reporting of population, intervention, and outcomes.

## Introduction

Language impairments are a common cognitive consequence of stroke and contribute to shrinking social networks and decreased quality of life (1, 2). Cognitive impairments, including language impairments, may also impact the ability to participate in and benefit from rehabilitation (3, 4). While behavioral interventions are the conventional, evidence-based approach to treating stroke-related cognitive deficits [e.g., (5)], they require considerable time and practice, and patients are often discharged prior to resolution of these difficulties (6). Accordingly, there has been growing empirical interest in adjuvant therapies, including exercise^1^, that can maximize behavioral intervention outcomes (10–15). The focus of the current systematic review is whether aerobic exercise alone affects language and other cognitive outcomes following stroke, which will help ascertain its potential effectiveness as an adjuvant to behavioral therapy in this population.

Several lines of evidence suggest that physical activity holds merit as a means to ameliorate post-stroke language and cognitive sequelae. First, the physical and mental health benefits (e.g., increased strength, reduced falls, mood enhancement, better cardiovascular health) associated with physical activity are well-established in typical and atypical aging populations (16–20). Though there are challenges in introducing aerobic exercise to stroke survivors (21), positive effects of physical activity on the physical sequelae of stroke (e.g., hemiparesis) have strong empirical support, and accordingly, exercise (e.g., range of motion exercises, strength training) is a recommended approach in several stroke management guidelines [e.g., (22, 23)]. Second, although results have been mixed depending on study design and choice of outcome measures (24, 25), it is generally recognized that physical activity is beneficial for cognitive performance in older adults who are healthy or experiencing cognitive decline, with recent data demonstrating an inverse relationship between physical activity and risk of cognitive decline (16, 26, 27). Further, increased cardiovascular fitness associated with physical activity, specifically aerobic exercise, has been shown to positively affect cognitive function in studies of healthy and cognitively-impaired individuals [e.g., (26, 28)]. Third, neural changes associated with repeated or long-term aerobic exercise include increased concentration of neurotrophic and growth factors (e.g., BDNF), which can induce cellular changes such as creation of glial cells, neurons, synapses, and blood vessels (16, 29, 30). These cellular changes allow for structural enhancements such as increased perfusion and gray/white matter volume, and in turn, result in increased brain activation and functional connectivity. Collectively, these molecular, cellular, structural, and functional changes support improved cognitive and motor function. Finally, in animal models, aerobic exercise has been shown to promote functional recovery following neurologic injury [see (30) for a review].

It follows that aerobic exercise would benefit cognitive functioning following stroke; accordingly, recent systematic reviews have concluded that aerobic exercise may enhance cognition in the stroke population [e.g., (30–36)]. However, most advocate for further examination of the effects of exercise on post-stroke cognitive abilities, identifying methodological issues and inconsistent outcomes across studies. Importantly, the focus and methods of previous systematic reviews have varied, with no particular attention given to the cognitive domain of language. Considering that language difficulties are a common consequence of stroke, addressing this gap in knowledge has immediate clinical applicability and thus language outcomes are examined in the current review.

Related to the limited examination of language abilities as a cognitive domain that may be responsive to exercise, there is a concern as to whether stroke survivors with aphasia have been represented in the extant literature. For example, the word “aphasia” was absent in previous systematic reviews of exercise and cognition post-stroke, [e.g., (35–37)]. This is surprising, given that approximately one-third of stroke survivors are living with aphasia (38, 39) and experience changes with both language and other cognitive abilities (40). Understanding the impact of aerobic exercise on individuals both with and without aphasia post-stroke is vital to informing rehabilitation for stroke survivors, their caregivers, and rehabilitation professionals, particularly speech-language pathologists who address language and other cognitive abilities.

The overall objective of the Aphasia Writing Group, a subset of the Evidence-Based Clinical Research Committee of the Academy of Neurological Communication Disorders and Sciences, was to conduct a systematic review to examine what is currently known about the utility of aerobic exercise for improving cognitive abilities, including language, in individuals affected by stroke and stroke-related aphasia. The specific aims were to:

- Characterize how aerobic exercise affects different areas of cognition after stroke, paying particular attention to language outcomes.
- Characterize commonalities and differences across studies with positive vs. null cognitive outcomes following aerobic intervention, including outcome measures, participant characteristics, and intervention characteristics.
- Ascertain the representation of persons with aphasia (PWA) in this domain of the stroke literature.

## Methods

### Search Strategy

**Figure 1:**
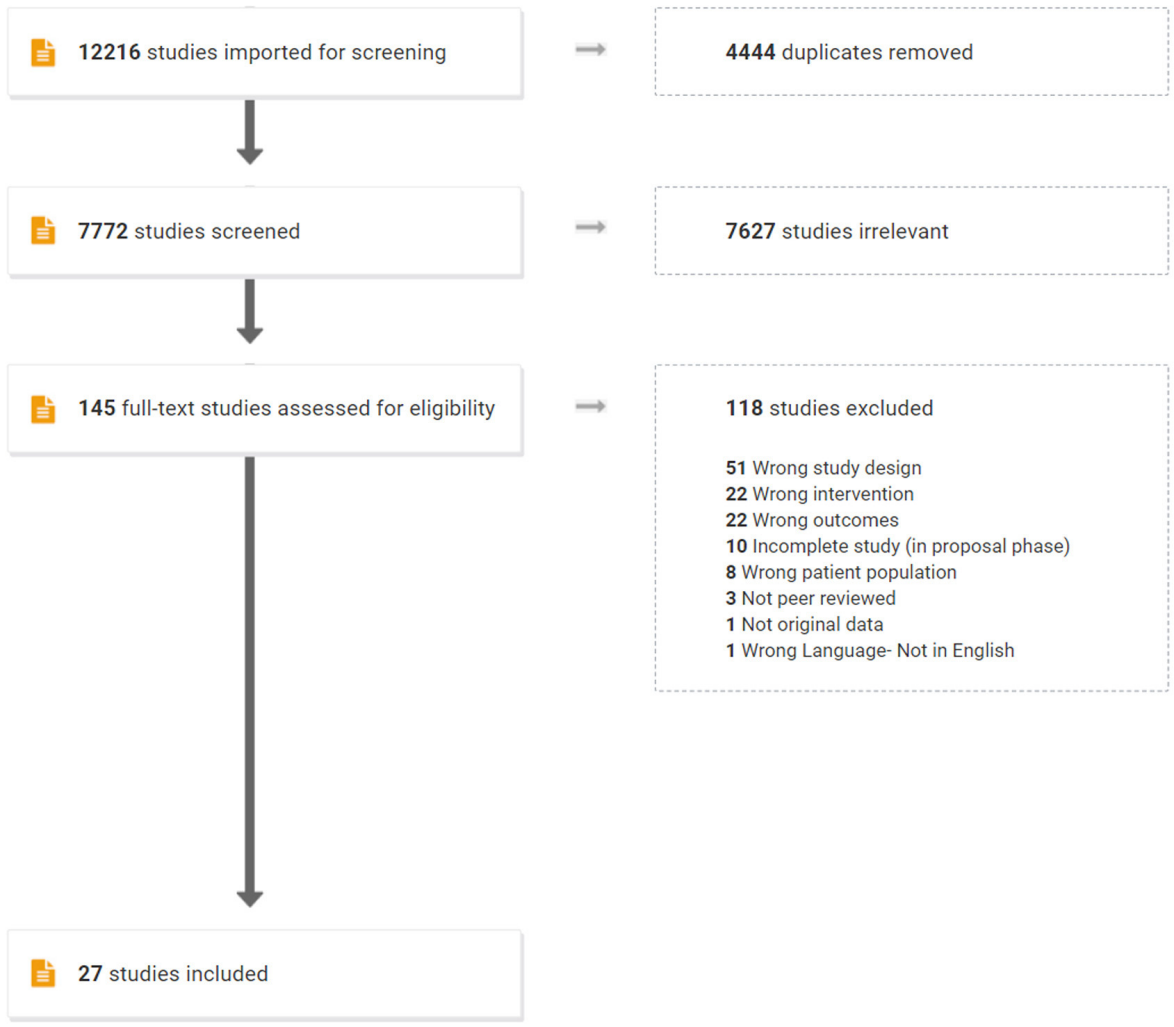
Prisma diagram generated from Covidence.

A comprehensive and systematic literature search was conducted from 2008 through September 2020 to gather state-of-the-art information. Following consultation of librarians from the home universities of the authors of this manuscript, the following databases were searched: Web of Science, CINAHL, PubMed, Medline, ProQuest, PsycInfo, COMDisDome, SpeechBite, ASHAWire, and Scopus. Reference lists of included studies were reviewed to identify any studies that did not emerge from the databases search. The search targeted peer-reviewed, clinical trials (i.e., a study aimed at evaluating an intervention) reporting use of an aerobic exercise intervention post-stroke to improve cognitive outcomes. Specifically, inclusion criteria included the following: full text peer-reviewed journal article in English; participants must have had a stroke and be 18 years or older; the intervention must be aerobic exercise without language or cognitive training; and outcomes must include language and/or other cognitive domains. Studies that included language or cognitive training along with aerobic exercise were excluded in order to evaluate the direct impact of aerobic exercise on cognition. Gray literature and non-experimental publications (e.g., reviews) were excluded. The Preferred Reporting Items for Systematic reviews and Meta-Analysis Guidelines [PRISMA; (41)] were followed. Search terms reflected categories of population (e.g., stroke, aphasia), intervention (e.g., aerobic exercise), and outcome (e.g., cognition, language). Terms within or across a category were combined via “OR” or “AND,” respectively (see Supplementary Table 1). This search yielded 7,771 articles after 4,444 duplicates were removed (Figure 1). Deduplication and screening were performed *via* Covidence, which uses PRISMA guidelines by default (https://www.covidence.org/).

The authors conducted a title and abstract review to manually scan for the inclusionary criteria of: original data from adults with stroke/CVA and no other neurological diagnoses, inclusion of an intervention group receiving aerobic exercise only, and measuring an outcome related to language and/or other cognitive domains. To examine reliability for this screening process, a randomly selected sample of 300 consecutive titles and abstracts was independently screened by one of the authors (LM). Inter-rater agreement was 98.7%, with disagreements resolved *via* discussion among all authors. A full-text review of each of the remaining 145 articles was then completed independently by two authors, resulting in 27 articles for inclusion. Inter-rater agreement for full-text review was 90.4% with disagreements resolved via discussion among all authors. Table 1 lists the 27 studies and the type of design of each study, which consisted of randomized and non-randomized clinical trials.

**Table 1:** Design and PEDro+ Score of included studies.

**Study#**	**References**	**Design**	**PEDro+ Score (max = 12)**
1	^*^Bo et al. (42)	RCT	8
2	Chan and Tsang (43)	RCT	8
3	^*^Colledge et al. (44)	non-randomized controlled trial	4
4	^*^ El-Tamawy et al. (45)	case-control study	6
5	^*^Ezeugwu and Manns (46)	prospective cohort	5
6	^*^Ihle-Hansen et al. (47)	RCT	7
7	^*^Khattab et al. (48)	RCT	9
8	^*^Kim and Yim (49)	RCT	7
9	^*^Kluding et al. (50)	prospective cohort	3
10	Krawcyk et al. (51)	RCT	8
11	^*^Lee et al. (52)	prospective cohort	4
12	Macko et al. (53)	prospective cohort	4
13	^*^Marzolini et al. (54)	prospective cohort	4
14	^*^McDonnell et al., (34, 55)	RCT	5
15	Meester et al. (56)	RCT	6
16	^*^Moore et al. (57)	RCT	9
17	^*^Moriya et al. (58)	prospective cohort	3
18	Pallesen et al. (59)	RCT	8
19	Ploughman et al. (60)	RCT crossover	6
20	Ploughman et al. (61)	RCT	10
21	Quaney et al. (62)	RCT	7
22	^*^Rosenfeldt et al. (63)	RCT	8
23	Stuart et al. (64)	non-randomized controlled trial	8
24	^*^Swatridge et al. (65)	interrupted time series	5
25	Tang et al. (66)	RCT	8
26	^*^ Unibaso-Markaida et al. (67)	non-randomized controlled trial	5
27	^*^Yoo and Yoo (68)	RCT	9

* *Studies that showed positive cognitive effects of aerobic exercise. RCT, randomized controlled trial. The study number in this table is used to identify these articles in the remaining tables*.

### Methodological Quality Review

Two reviewers independently performed a quality assessment of each included article. Inter-rater agreement prior to consensus was 87%. The quality assessment involved a “risk of bias comparison” based on the Physiotherapy Evidence Database Plus (PEDro+) Scale (69), which includes 13 criterion items with a maximum score of 12 (see Supplementary Table 2 for a full description of the PEDro+). The PEDro+ scale features two additional criteria that are critical elements of behavioral treatment (Treatment Fidelity and Treatment Replicability), which are not present on the original PEDro scale. The PEDro and PEDro+ scales are commonly used in the stroke rehabilitation literature to evaluate the methodological quality of randomized controlled trials; however, these scales are also often used to evaluate other types of clinical trials, including non-randomized controlled trials and case series research designs (69–74). For these reasons, the PEDro+ scale was deemed a valid and appropriate appraisal tool to evaluate the 27 clinical trials reviewed in this study.

### Data Extraction

Data related to the specific aims were extracted from each article by two reviewers. Inter-rater agreement prior to consensus was 96%. These data included specifics on study design, participant characteristics (e.g., age, time, post-stroke onset), exercise intervention (e.g., type, frequency), cognitive outcome measures (e.g. self-reported, performance-based), and results pertaining to those outcome measures.

### Identification of Outcome Measures and Cognitive Domains Tested

The language and other cognitive outcome measures used in each study were collated based on whether they were self-reported or quantitative/performance-based and what domain(s) of cognition they measured. Supplementary Table 4 lists each outcome measure, its cognitive domain(s), and each study that included the measure. The self-report measures—SIS (75) and Stroke Specific Quality of Life Scale [SS-QOL; (76)]—both include domains related to communication and thinking and were thus included in the counts as measures of language and executive function.

## Results and Discussion

### Methodological Quality

None of the 27 included studies received the maximum score of 12 on the PEDro+ scale (69). Across studies (Table 1), total scores ranged from 3 [high bias for nine items, e.g., (50)] to 10 [high bias for two items, e.g., (61)] with an average score of 6.39. It should be noted that if a PEDro+ item was not explicitly addressed, then it received a high bias rating and “0” score. Therefore, scores might reflect the report available to the reader rather than the procedures implemented by the authors. See Supplementary Table 3 for PEDro+ scale item-level detail per study.

**Figure 2:**
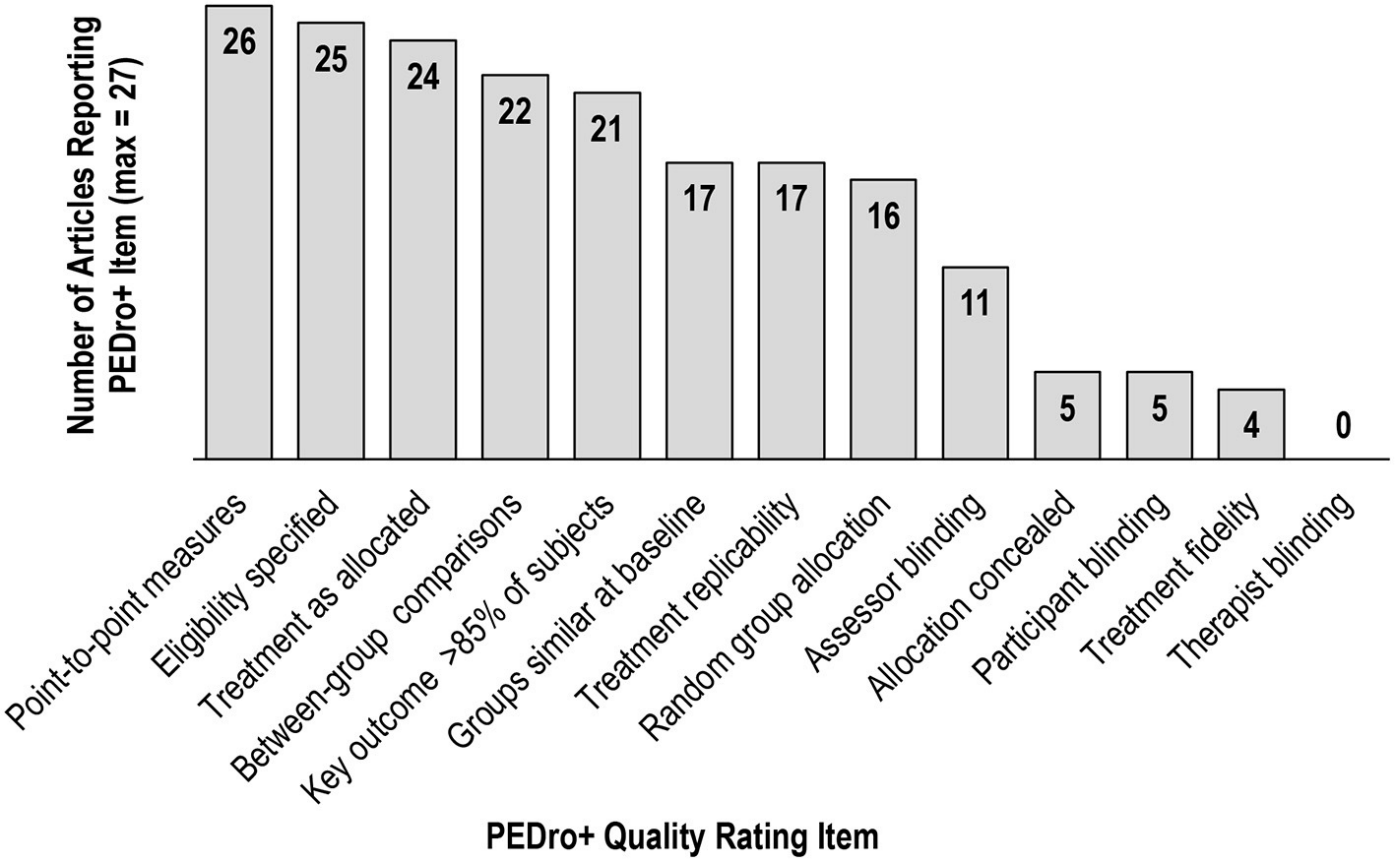
PEDro+ Scale item reporting.

Fourteen of the 27 studies received a low bias judgment on more than half of PEDro+ items, suggesting they were of high quality (69). However, as shown in Figure 2, there was variation in terms of which PEDro+ items received low bias ratings across studies. High bias judgments were common for Allocation Concealment, Treatment Fidelity, and Blinding of Assessors, Subjects, and Therapists, with all studies lacking Therapist Blinding. De Morton (71) reported that Therapist and Subject Blinding criteria had the least adherence in her Rasch analysis of 200 clinical trials of various intervention types that were randomly selected from the PEDro database (www.pedro.org.au). De Morton explained blinding is often difficult or not possible in many clinical intervention trials. This logistical dilemma likely applied to some of the studies included in this review and should be considered when evaluating the quality scores.

Notably, studies with only one exercise group cannot satisfy the Therapist Blinding, Subject Blinding, nor Allocation Concealment criteria due to the nature of their design. Therefore, the seven single group studies lacking a comparison or control group received lower PEDro+ scores, highlighting these types of clinical trials are not as methodologically rigorous compared to clinical trials with random assignment of multiple groups. However, it is important to note that the PEDro+ score does not indicate study meaningfulness, treatment effect size, or generalizability (69), and studies with lower PEDro+ scores might perform well in those areas.

The Assessor Blinding and Treatment Fidelity PEDro+ items were applicable to all study designs included in this review, and the low reporting incidence of these items suggests these criteria warrant more careful consideration when designing and publishing future investigations. Assessor blinding is critical to obtain unbiased performance on outcome measures pre- and post-treatment, and reporting of treatment fidelity is essential for confirming valid, reliable treatment implementation.

Similar to previous systematic reviews focused on exercise and cognition after stroke [e.g., (34, 37, 77)], the quality of papers included in the current review was wide ranging, indicating more rigorous study design and reporting are needed to further strengthen this area of the stroke literature. Given the variation in methodological rigor and consistent under-reporting on several PEDro+ items across studies, results pertaining to the cognitive benefits of exercise subsequent to stroke should be interpreted with caution.

### Outcome Measures and Cognitive Domains

**Figure 3:**
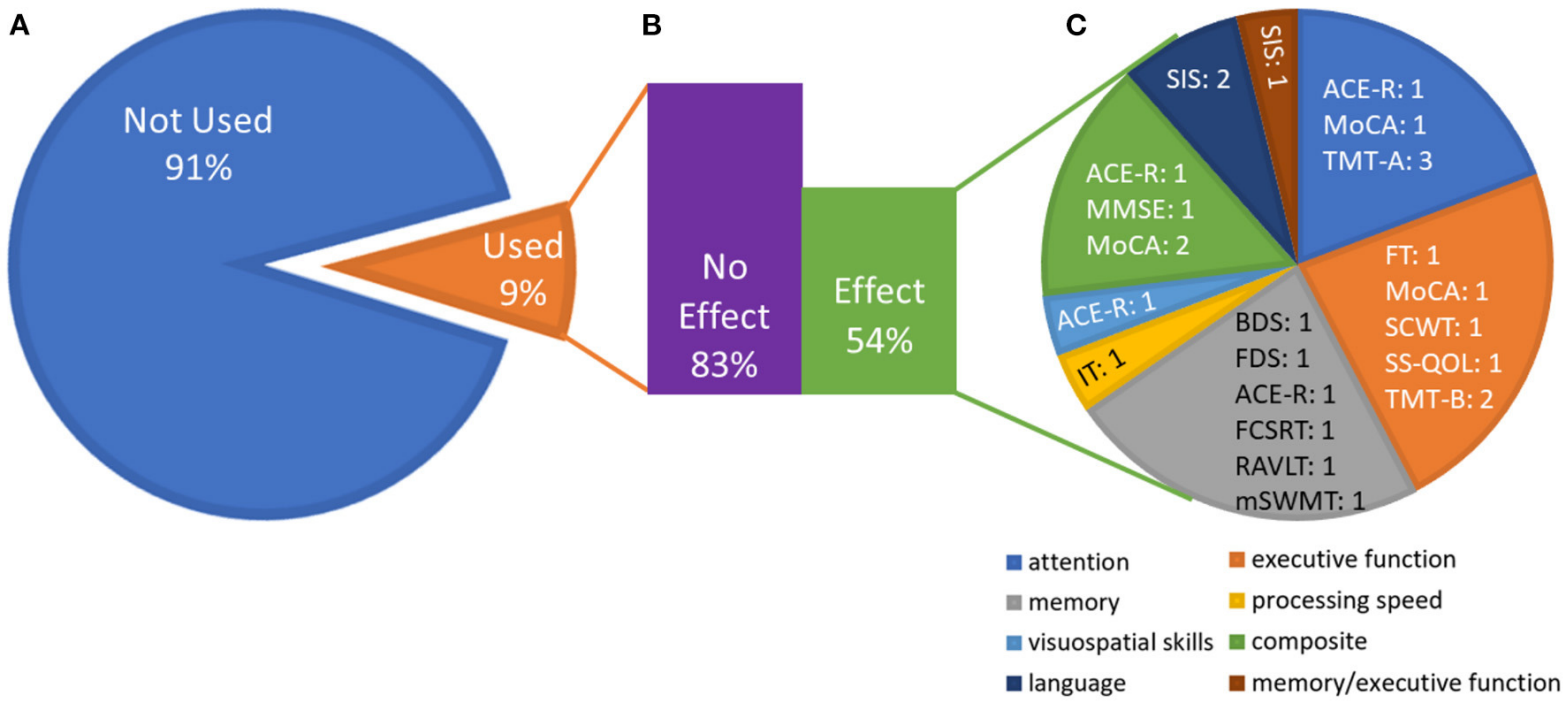
Effects broken down by outcome measures and cognitive domains. **(A)** Proportion of studies that utilized the outcome measures identified in this review, averaged across outcome measures (e.g., the Stroop task was included in 6 of the 27 studies). This emphasizes the variability in the outcome measures chosen across studies. **(B)** Average (across outcome measures) of the proportion of studies that used the outcome measure and showed an effect vs. no effect (e.g., among the six studies using the Stroop task, five showed no effect). The numbers do not add up to 100% because many measures were only reported in one study. **(C)** Domains of outcome measures that showed an effect. Each domain contains measures that contribute to that domain and the number of studies that showed an effect on that measure. The “composite” domain represents effects for which only the composite score on cognitive screeners was reported. See Supplementary Table 4 for test abbreviations.

Although cognition was not the primary focus of nearly half of the 27 included studies [which instead prioritized a physical outcome; e.g., (46, 51, 52, 57)], each reviewed study included some measure of cognition and was thus deemed appropriate for data extraction. A description of the cognitive outcome measures found in the studies reviewed is listed in Supplementary Table 4. An illustration of the inclusion of cognitive measures across studies and whether or not an effect was noted is included in Figure 3.

Overall, seven studies included a self-report measure and 23 included at least one performance-based cognitive measure. Fifteen studies included at least one measure of language, 20 included at least one measure of attention, 19 included at least one measure of memory, 20 included at least one measure of executive function, and 12 included at least one measure of visuospatial skills. Four studies used only a self-report measure of cognition [SIS, SS-QOL: (53, 63, 64, 68)]. Nine studies utilized cognitive screeners (MMSE, MoCA, or ACE-R), which offer a cursory assessment of a broad range of cognitive functions; two of these studies reported on specific domains tested by the cognitive screener (51, 56). Two studies that used a cognitive screener also included a self-report measure [SIS: (46, 57)], and two included additional tests of more specific cognitive abilities [TMT A and B: (47, 49) and Stroop Color and Word Test (SCWT): (49)]. Sixteen studies included performance-based tests of specific cognitive abilities, five of which focused specifically on one cognitive domain (43, 44, 58, 61, 65). Six studies specifically reported the results from language subtests of screeners or self-report measures (50, 51, 53, 56, 59, 64). No study used a language-specific, performance-based measure. Seven studies quantitatively measured all of the following cognitive domains: attention, memory, executive functioning, visuospatial skills, and language. Of these, three reported the effect of exercise on each domain separately (51, 56, 59).

Interestingly, no study used a full battery of standardized cognitive testing, even in studies focused specifically on cognitive outcomes of aerobic exercise after stroke. Relatedly, the rationale for the selection of cognitive measure(s) was infrequently provided. Relevant to our particular interest in language outcomes, no study used a performance-based measure that focused solely on evaluating language abilities, with language assessment being primarily limited to brief spoken naming tasks. Therefore, whether aerobic exercise can positively influence the broad range of language abilities that may be compromised by stroke, particularly those abilities enmeshed in daily communication activities (e.g., discourse comprehension; grammatical skills germane to spoken or written sentence formulation), has not yet been examined.

### Aim 1: Effect of Aerobic Exercise on Cognition

As shown in Tables 2 and 3, of the 27 studies reviewed, 10 reported no statistically significant improvement in any cognitive domain. Of the 17 studies that showed improvement in at least one cognitive domain, only two reported a significant increase in language as measured *via* the communication domain of the SIS (46, 63). Six studies reported a significant increase in memory (44, 45, 50, 58, 59, 67), five reported a significant increase in executive function (42, 49, 54, 65, 67), five reported a significant increase in attention (47, 49, 54, 67), and one reported a significant increase in visuospatial skills (45). Additionally, one study reported improvement on the memory/thinking portion of the SIS (50), and one study reported improvement on the thinking portion of the SS-QOL (68). Four studies only reported the overall score on a broad cognitive screener (46, 49, 52, 57), making it difficult to determine which specific cognitive functions improved in these studies. Notably, no study identified a significant decrease in specific or general cognitive abilities subsequent to aerobic exercise.

**Table 2:** Participant characteristics in reviewed studies.

**Study#**	**N**	**Age M**	**Sex M/F**	**TPO M (SD) in months**	**Stroke characteristics**
**Studies with positive cognitive effects**					
1	42	65.12	23/19	>6	NR
3	15	57.3	4/11	44 (28.9)	SAH; 4 RMCA, 3 LMCA, 3 RpComm, 3 LaComm, 1 RaComm
4	15	48.4	NR	3-18	anterior ischemic stroke
5	34	64.6	19/15	3.5 (1.1)	19 RH, 15 LH; 26 ischemic, 8 hemorrhagic
6	177	71.4	99/78	NR	163 infarction; 14 hemorrhage
7	22	65.9	13/9	39.6 (48.0)	15 RH, 10 LH; 3 lacunar, 7 ischaemic, 9 hemorrhagic, 6 unknown
8	14	50.71	9/5	12.79 (7.34)	NR
9	9	63.7	5/4	50.4 (37.9)	3 RH, 6 LH
11	28	56.74	all M	26.04 (19.08)	8 RH, 20 LH; all ischemic
13	41	63.6	30/11	>10 weeks	65.9% ischemic
14	17	70.1	12/5	106.8 (CI = 67.2, 146.4)	52% LH; 82% ischemic
16	20	68	18/2	21 (37)	10 RH, 9 LH, 1 bilateral; 1 hemorrhagic; 5 cortical, 4 BG, 2 cerebellar, 7 subcortical other, 2 unknown
17	11	69.6	7/4	NR	5 ischemic, 6 hemorrhagic
22	32	55.5	22/10	14 (average median)	NR
24	9	57.8	6/3	37.6 (23)	7 RH, 2 LH; 6 ischemic, 1 ischemic and hemorrhagic (2 unreported)
26	15	58.43	10/5	1-12	11 RH, 4 LH; 10 ischemic, 5 hemorrhagic
27	28	60.86	17/11	66.6 (21.6)	17 RH, 11 LH
**Studies with null cognitive effects**					
2	17	62.7	10/7	93.6 (73.2)	7 RH, 10 LH; 11 ischemic, 5 hemorrhagic, 1 both
10	31	63.7	23/8	NR	19 RH, 12 LH; lacunar: 17 BG, 9 thalamus
12	20	70	9/11	56 (22)	NR
^*^15	21	62.25	11/10	25.71 (32.70)	13 RH, 6 LH, 5 midbrain; 13 ischemic, 10 hemorrhagic, 1 both
18	16	55	9/7	NR	6 RH, 8 LH, 2 (unknown); 13 CVA, 3 SAH
19	21	61.4	13/8	20.1 (14.6)	19 ischemic, 2 hemorrhagic; 6 cortical, 11 subcortical, 3 both, 1 brainstem
^*^20	13	58.4	9/4	36.0 (53.4)	6 RH, 6 LH, 1 bilateral; 12 ischemic, 1 hemorrhagic
21	19	64.1	10/9	55.44 (38.52)	all ischemic
23	40	66.6	25/15	50.4 (9.6)	26 RH, 14 LH
25	25	66	14/11	42 (range: 26.4-80.4)	15 RH, 10 LH; 3 lacunar, 7 ischemic, 9 hemorrhagic, 6 unknown; 4 cortical, 7 subcortical, 5 brainstem, 9 unknown

* *Studies that included PWA; NR, not reported; RH, right hemisphere; LH, left hemisphere; CVA, cerebrovascular accident; PWA, persons with aphasia; SAH, subarachnoid hemorrhage; BG, basal ganglia; aComm, anterior communicating artery; pComm, posterior communicating artery*.

**Table 3:** Intervention characteristics in studies reviewed.

**Study#**	**Group vs. individual**	**Exercise type**	**Intensity**	**Intensity definition**	**Activity**	**Supervision**	**Session duration x frequency**	**Total min/week**	**Program duration**
**Studies with positive cognitive effects**									
1	Group	Aerobic + Resistance	Moderate	BPES 13-15	jogging, cycling, strength, balance	Supervised	50 m ×3 d/w	150	12 w
3	Individual	Aerobic	Moderate to High	55-85% HRmax	walking	Both	30-45 m ×3-5 d/w	90-225	12 w
4	Group	Aerobic + Resistance	NR	NA	PT + cycling	Supervised	30 m PT + 45 m aerobic ×3 d/w	135	8 w
5	Individual	Aerobic	NR	6,000+ steps/day	STUFFS, walking	Unsupervised	NR	NR	8 w
6	Individual	Aerobic	Goal: High	Self-report	individualized: any physical activity 30 m/d + “vigorous” activity 2-3 d/w	Unsupervised	30 m/d active + 45-60 m vigorous ×2-3 d/w	90-180	72 w
7	Group	Aerobic	Moderate to High	40-80% HRmax	walking, cycling, marching, sit-to-stand, platform steppers	Supervised	60 m ×3 d/w	180	24 w
8	NR	Aerobic + Resistance	NR	NA	handgrip exercise + walking (treadmill)	Supervised	15 m strength + 20 m walking ×5 d/w	100	6 w
9	Group	Aerobic + Resistance	Light to Moderate	50% VO2max	TBRS + resistance training (bands) of lower extremities	Supervised	60 m ×3 d/w	180	12 w
11	Group	Aerobic + Resistance	Moderate	60% VO2reserve	cycling + strength	Supervised	55 m aerobic ×3 d/w + strength ×2 d/w	165	24 w
13	Both	Aerobic + Resistance	Moderate	40-70% HRreserve/ VO2max; BPES 11-16	walking, stationary recumbent, upright cycling	Both	20-60 m ×5 d/w	100-300	24 w
14	Group	Aerobic + Resistance	NR	NA	aerobic circuit class + resistance training	Supervised	60 m ×1-2 d/w	60-120	24 w
16	Group	Aerobic + Resistance	Light to Moderate	40-80% HRmax	FAME: strength, balance, walking/jogging, marching	Supervised	45-60 m ×3 d/w	135-180	19 w
17	NR	Aerobic	Moderate	40% VO2max	Ergometer	Supervised	15 m ×1 session	NA	NA
22	Group	Aerobic + Resistance	Moderate to High	60-80% HRreserve	recumbent cycling + UE-RTP	Supervised	45 m ×24 sessions	NR	NR
24	NR	Aerobic	Moderate	45-55% HRreserve	semi-recumbent stepper	Supervised	20 m ×1 session	NA	NA
26	Group	Aerobic	NR	NA	Nintendo Wii Sports Resort: archery, tennis, golf, bowling, cycling, air sports	Supervised	30 m ×3 d/w	90	8 w
27	Group	Aerobic + Resistance	NR	NA	walking + functional UE tasks (theraband, armcycle, lifting weighted box)	Both	90 m ×3 d/w	270*	24 w
**Studies with null cognitive effects**									
2	Group	Aerobic + Resistance	NR	NA	walking + seated stretching and strengthening	Supervised	60 m ×2 d/w	120	12 w
10	Individual	Aerobic	High	77-93% HRmax; BPES 14-16	Home-based HIIT, self-chosen exercise modality; provided stationary bike if needed	Unsupervised	3 ×3 m + 2 m “active recovery,” 5 d/w	55	12 w
12	Group	Aerobic	NR	NA	walking with handrail support, weight-shifting, ROM	Supervised	60 m ×2 d/w	120	8 w
15	Individual	Aerobic	Moderate to high	55-85% HRmax	treadmill walking	Supervised	45 m ×2 d/w	90	10 w
18	Group	Aerobic	Moderate to high	>70% HRmax	cycling, platform stepper, sit-to-stand	Supervised	50 m ×2 d/w	100	4 w
19	NR	Aerobic	Moderate	70% HRmax	treadmill walking	Supervised	20 m ×2 sessions	NR	NR
20	Group	Aerobic	Light to moderate	60% VO2max	treadmill walking	Supervised	60 m ×3 d/w	180	10 w
21	Group	Aerobic	Moderate	70% HRmax	stationary bike	Supervised	45 m ×3 d/w	135	8 w
23	Group	Aerobic + Resistance	NR	NA	APA: walking, strength, balance	Supervised	60 m ×3 d/w	180	24 w
25	Group	Aerobic	Moderate to high	40-80% HRreserve	individualized aerobic training (modality NR)	Supervised	60 m ×3 d/w	180	24 w

*NR, not reported; NA, not applicable; BPES, Borg Perceived Exertion Scale; STUFFS, Stand up Frequently from Stroke; PT, physical therapy; TBRS, Total Body Recumbent Stepper; FAME, Fitness and Mobility Exercise Program; UE-RTP, upper extremity repetitive task practice; HIIT, High Intensity Interval Training; ROM, range of motion; APA, Adaptive Physical Activity stroke program; HRmax, maximum heart rate; HRreserve, heart rate reserve (difference between maximum and resting heart rate); VO2max, maximal oxygen uptake; VO2reserve, oxygen uptake reserve (difference between maximum and resting oxygen uptake); d/w, days per week; m, minutes*.

It is difficult to derive strong conclusions regarding the specific cognitive effects of aerobic exercise for stroke survivors for the following reasons: (a) the heterogeneity of domain-specific measures, (b) the use of cognitive screeners—which may not be suitable for identifying nor measuring change in single domain strengths and weaknesses (78, 79), (c) the use of self-report as the sole measure of cognitive outcome, and (d) the reliance on subtests of cognitive screeners and self-report measures to assess language abilities. This is not entirely unexpected given that cognitive outcomes were often not the focus of the studies. However, the modest cognitive effects in concert with methodological variability and study quality concerns as identified in the current review, were previously reported by Zheng et al. (37), indicating little progress in this aspect of the literature in the past 5 years. Thus, there is a clear opportunity for future studies to address these concerns.

### Aim 2: Commonalities Across Studies

Substantial variability in outcome measures, and both variability and reporting issues regarding participant profiles and intervention characteristics across studies made it difficult to identify similarities among the studies showing positive vs. null cognitive effects of exercise. With respect to cognitive outcome measures, the TMT was most commonly used; yet, just 2 of the 10 studies that utilized this measure reported statistically significant improvement (42, 67). This either suggests that the TMT B is not sensitive to the effects of aerobic exercise, or that aerobic exercise does not improve executive function, though other measures of executive function did show improvement across studies (see section Aim 1: Effect of Aerobic Exercise on Cognition and Figure 3). The next most common measures were utilized in 5-6 studies and included the TMT A, forward and backward digit span, the Stroop task, Stroke Impact Scale, and the MoCA. Of these, the only measure that showed more positive than null effects among studies that used the same measures was the MoCA, with three studies (out of five) reporting improvements (46, 49, 54). Again, this either suggests that the TMT A, forward and backward digit span, the Stroop task, and the Stroke Impact Scale are not sensitive to the effects of aerobic exercise or that exercise does not improve attention, short term memory, working memory, inhibition, and self-reported cognition and communication, respectively. However, the MoCA, and other measures of attention, memory, and communication, did show improvement across studies (see section Aim 1: Effect of Aerobic Exercise on Cognition and Figure 3). Notably, only Marzolini et al. (54) specifically reported whether the MoCA improvement was meaningful [i.e., performance changed from impaired to unimpaired; (80)], which is important, given that the purpose of the MoCA is solely to detect the presence or absence of cognitive impairment (81).

With respect to participant characteristics, range of average participant age for studies reporting positive effects of aerobic exercise on cognition was 48-70 years, whereas the range for studies reporting null effects was 58-70 years; the average age across participants appeared similar in the positive and null outcomes studies (61.6 vs. 63 years, respectively). Regarding time post-onset (TPO), studies with positive effects had a range of 1-66 months, while those with null effects all included individuals who were at least 2 years TPO (note two studies in each of the positive and null outcomes categories failed to include any TPO information). While spontaneous recovery could have contributed to the gains seen in the 17 studies with positive effects, 11 of these studies solely included individuals more than 2 years TPO, suggesting that aerobic exercise is associated with cognitive change in the chronic stage (i.e., more than 6 months post-stroke). Nonetheless, TPO may be an important factor to examine in future work.

Regarding intervention characteristics, across all studies, exercise intensity (i.e., level of effort) and modality (e.g., cycling, walking, stepping) were highly variable, with no discernable pattern differentiating studies reporting positive vs. null cognitive outcomes. Of note, five positive and three null outcome studies failed to report any information regarding exercise intensity, and the remaining studies defined and monitored intensity levels in a variety of different manners (e.g., relative to maximal heart rate, maximal oxygen consumption, or rating of perceived exertion), mirroring a general lack of consensus across the stroke rehabilitation literature (82). Likewise, no patterns emerged for studies reporting group vs. individual exercise programs, nor supervised vs. unsupervised exercise sessions, supporting the idea that the beneficial effects of exercise are dissociated from the social engagement inherent in group settings [e.g., (83)]. Visual inspection of the studies reporting positive cognitive outcomes revealed that a substantial proportion (60 vs. 20% of null outcome studies) utilized a combination of aerobic exercise and strength training, consistent with evidence in the healthy aging literature of a positive and possibly synergistic effect of aerobic exercise combined with strength training regimens (83–85). Additionally, those studies with positive cognitive outcomes tended to provide exercise programs more frequently (3-5 days/week) and for a longer overall duration (at least half lasting for longer than 19 weeks), compared to studies with null effects. Indeed, it has been suggested that fitness programs for older adults are more likely to engender positive cognitive effects when implemented for 6 or more months (66, 86), leading some to conclude that cognitive changes following exercise programs are time- rather than intensity-dependent, particularly for stroke survivors (85).

Taken together, our search for commonalities across studies to guide future incorporation of aerobic exercise into post-stroke and aphasia management yielded little definitive information due to a combination of factors, including a diversity of cognitive assessment measures and lack of consideration of clinically vs. statistically significant change across studies, and underreporting of participant and intervention characteristics. Although trends supporting possible best practices for evoking cognitive effects were found (e.g., pairing aerobic exercise with resistance training, providing intervention 3 or more days per week), these patterns were not robust nor detailed enough to guide clinical practice without further study. Enhanced reporting of exercise interventions, including greater detail regarding protocol and dosage, in future work will allow for better comparison across studies and replication to validate findings.

### Aim 3: Representation of PWA

Across the 27 reviewed studies, nine had no mention of including or excluding PWA. In the remaining 18 studies, 15 reported inclusionary and/or exclusionary criteria that directly [e.g., exclusionary criterion of “severe aphasia” (53), p. 324] or indirectly [e.g., exclusionary criterion of “…inability to follow 1-2 step commands” (63), p. 924] pertained to aphasia, only two of which (59, 61) provided information regarding how many of their participants did or did not have aphasia. The other three studies referring to aphasia (48, 56, 66) indicated consideration of PWA in the absence of any aphasia-related inclusionary/exclusionary criteria. For example, Khattab et al. [(48), p. 3] reported excluding the data of four participants (two with “significant aphasia,” two with “difficulty understanding test instructions”) who completed the aerobic exercise intervention but had incomplete cognitive outcome data. Khattab et al. (48) did not, however, specify if any remaining participants had less “significant” aphasia symptoms. Similarly, Tang et al. [(66), p. 843] stated that cognitive test data were missing for four participants due to “significant aphasia” and one participant due to “difficulty understanding test instructions;” they also stated that “for those with missing pre-training data points, these participants were excluded from the analysis.” Because (a) Tang et al. (66) did not explicitly state whether the participants with significant aphasia were in their aerobic exercise vs. control balance training group, (b) it is not clear in their cognitive outcome table which participants are linked to the missing data (e.g., participants with significant aphasia vs. comprehension issue), and (c) these researchers did not specify if any of their other participants had less “significant” aphasia, it is difficult to determine if there was any aphasia representation in their study. Furthermore, those studies directly or indirectly excluding those with severe PWA often failed to report whether any participants did present with mild-to-moderate aphasia or another cognitive-communication disorder. Across the 18 studies that made some mention of aphasia or acquired language difficulties, there was nominal information (e.g., no mention of what aphasia test was used or what healthcare professional made the aphasia diagnosis) about how the presence and severity of aphasia was determined.

In total, only three reviewed studies (56, 59, 61) detailed how many participants had or did not have aphasia. There was a total of 12 PWA across these studies who completed aerobic exercise intervention and whose cognitive test data were used in analyses. Meester et al. (56) specified including six participants with mild aphasia but did not specify how mild aphasia was identified. Ploughman et al. [(61), p. 204] also included six participants with expressive aphasia (“mild-severe”) in their two participant groups (three in each), stating that the National Institutes of Health Stroke Scale (87) was used to identify expressive aphasia but failing to report aphasia severity from the Best Language item of this rating scale. McDonnell et al. (55) stated that individuals with severe expressive or receptive aphasia were excluded from their study on the basis of interviews, and that none of their participants had expressive aphasia, but neglected to specify who completed those interviews, how the absence of expressive aphasia was confirmed, nor whether any participants had mild or moderate receptive aphasia.

Accordingly, it is challenging to ascertain representation of PWA within the 27 included studies. A third of the studies offered no information about the aphasia status of participants, and the vast majority of studies that reported inclusionary/exclusionary criteria related to aphasia failed to specify if such criteria resulted in inclusion of PWA. Only three studies (56, 59, 61) provided sufficient information to determine how many of their participants had aphasia; however, for the only 12 PWA in these studies whose data were utilized, aphasia characteristics were not properly reported and the described aphasia assessment procedures were inadequate. Without knowing these participants' aphasia profiles, it cannot be determined whether their language symptoms confounded performance on cognitive measures. Additionally, none of these studies reported language outcomes. Such deficient reporting of aphasia has been identified in previous systematic reviews of the stroke literature [e.g., (88)] and more broadly in stroke rehabilitation studies (89), and is problematic given the prevalence of stroke-related aphasia (38, 39). Given our findings regarding aphasia representation within the included studies, it is not yet possible to determine if exercise can positively affect the cognitive symptoms of individuals with stroke-related aphasia.

There are methods to foster the participation of PWA in future exercise intervention research. Modifications to physical home exercise programs to reduce cognitive and language load such as simplifying syntax and vocabulary in instructional text, adding supportive images (personalized photographs of the participant performing each step may be particularly helpful), and using supportive communication techniques to maximize auditory comprehension and verbal expression have been used successfully to ensure program adherence in the context of aphasia (90, 91). Indeed, Marsden et al. (92) indicated that they had not used aphasia as an exclusionary criterion to assure that a broad spectrum of stroke survivors could participate and that they used “aphasia-friendly” [(92), p. 341] written material and presentation when providing education about physical activity and other aspects (e.g., goal-setting, fatigue management) of their community-based group intervention for stroke survivors and their caregivers. Nonetheless, Marsden et al. did not specify how many of their participants had aphasia, did not describe the “aphasia-friendly” modifications within the education component of their intervention, nor indicate if during the physical activity training component of their intervention, staff members who were supervising the activity sessions utilized supportive communication techniques. In studies including PWA and/or individuals needing cognitive or language supports, modifications to protocol instructions and materials to reduce cognitive and language load should be clearly detailed (91).

### Study Limitations

It is important to acknowledge limitations of the current review that may temper confidence in its findings. Only peer-reviewed articles published in English were considered. Because of the publication bias toward studies yielding positive findings, there may be studies yielding null findings in the gray literature that were missed. Because this review was intended to provide state-of-the-art information, only articles from 2008 and later were included; thus, the time-bound nature of this type of review may not capture the whole picture of the development of this subject area.

## Conclusion and Future Directions

Although results were mixed, this systematic review found some evidence for positive, direct effects of aerobic exercise on attention, memory, executive function, visuospatial skills, processing speed, and language after stroke (see Figure 3); however, methodological variability across studies limited our ability to identify which factors drove positive or null effects. Importantly, our review highlighted four primary areas to address in future research. First, individuals with aphasia were often excluded. As this represents approximately one-third of the stroke population (38), the inclusion of individuals with aphasia will help make the findings more generalizable. Second, a number of studies relied on cognitive screening measures (e.g., MMSE) that are not suitable for identifying nor measuring change in single domain strengths and weaknesses (78, 79). Thus, future work should include cognitive measures that are most appropriate for measuring change to improve sensitivity and reproducibility [see (55)]. Third, there was an absence of performance-based measures of language. Including performance-based language measures that can capture deficits often encountered in both right and left hemisphere strokes will help with a more complete understanding of the potential benefits of exercise in this population. Finally, much of the extant literature included cognitive measures that rely heavily on intact language for accurate measurement of the cognitive domain being tested (40, 93). For example, it is impossible to selectively measure executive function using a semantic fluency task with an individual who has anomia. Additionally, auditory comprehension difficulties may negatively affect performance on cognitive tests with complex instructions. Including non-linguistic cognitive measures that are accessible to stroke survivors who have difficulty with language will improve the interpretability of the results of exercise studies in this population.

Given the preliminary evidence that exercise improves cognition across multiple healthy and neurogenic populations [e.g., (13)], future studies are highly warranted for those with language and other cognitive sequalae following stroke. Future work should continue examining the direct impact of aerobic exercise on cognition post-stroke, as well as the combined effects of aerobic exercise and cognitive interventions for stroke survivors (12), particularly individuals with aphasia.

## Data Availability Statement

The original contributions presented in the study are included in the article/Supplementary Material, further inquiries can be directed to the corresponding author/s.

## Author Contributions

All authors contributed equally to the manuscript, including review conception, database search, data extraction, and manuscript writing. All authors have read, revised, and approved the submitted version of the manuscript.

## Funding

Sources of support included the Academy of Neurological Communication Disorders and Sciences and Northern Illinois University.

## Conflict of Interest

The authors declare that the research was conducted in the absence of any commercial or financial relationships that could be construed as a potential conflict of interest.

## Publisher's Note

All claims expressed in this article are solely those of the authors and do not necessarily represent those of their affiliated organizations, or those of the publisher, the editors and the reviewers. Any product that may be evaluated in this article, or claim that may be made by its manufacturer, is not guaranteed or endorsed by the publisher.

## Footnotes

^1^For the purposes of this paper, we will use the following definitions for physical activity, exercise, aerobic exercise, and strength/resistance training. We attempt to preserve terms used by individual studies, but use a more general term when different terms are used across studies. Physical activity: movement to increase energy expenditure above resting level (7). Exercise: prearranged, regulated and repetitive physical activity (7). Aerobic exercise: “exercises [that] demand oxygen without producing an intolerable oxygen debt, so they can be continued for long periods” [(8), p. 39]. Strength/resistance training: “a form of periodic exercise whereby external weights provide progressive overload to skeletal muscles in order to make them stronger” [(9), p. 208].
