# Supplementary material for: Cognitive and Linguistic Benefits of Aerobic Exercise: A State-of-the-Art Systematic Review of the Stroke Literature
Source: Front Rehabil Sci. 2021 Dec 24;2:785312. doi: 10.3389/fresc.2021.785312 (PMC9397720; doi:10.3389/fresc.2021.785312)
Supplement: Supplementary file 1 [file Table_1.DOCX]

**Supplemental Material**

**Table S1**

*Search Terms*

| **Key Element** | **Criteria** |
| --- | --- |
| Population | INCLUDE: stroke OR poststroke OR "post-stroke" OR ischemi* OR infarct OR "brain infarction" OR "cerebrovascular accident" OR CVA OR "cerebral artery occlusion" OR "intracerebral haemorrhag*" OR "subarachnoid haemorrhag*" OR "cerebral vascular accident" OR aphasia  EXCLUDE: rat OR mouse OR animal OR mice OR primate OR child* OR pediatric OR adolescent |
| Intervention | INCLUDE: aerobic exercise" OR "aerobic activity" OR "physical exercise" OR "physical activity" OR fitness OR "fitness training" OR "physical conditioning" OR "cardiovascular exercise" OR sports OR "exercise therapy" OR walking OR running OR treadmill OR "strength training"  EXCLUDE: Aerobic exercise paired with another therapy (i.e., speech-language therapy, cognitive therapy, social therapy) |
| Outcome Measure | INCLUDE: cognition OR language OR speech OR "word finding" OR anomia OR "word retrieval" OR communication OR learning OR "executive function" OR memory OR attention OR recovery OR plasticity OR neuroplasticity OR "mental process" OR performance OR neurotrophic |
| Study Design | INCLUDE: peer reviewed articles, experimental designs, empirical studies  EXCLUDE: reviews and meta-analyses, dissertations, conference proceedings or reports, published study proposals, editorials, non-peer reviewed journal articles. |
| Language | INCLUDE: English language journal articles only |

**Table S2**

*PEDro+ Items*

| **Item #** | **Item Name** | **Criterion** |
| --- | --- | --- |
| NR | Eligibility specified | Key participant characteristics for inclusion were described |
| 1 | Random group allocation | Participants randomly assigned to group or condition |
| 2 | Allocation concealed | Person determining eligibility is unaware of group assignment |
| 3 | Groups similar at baseline | Groups are similar on key variables at baseline |
| 4 | Participant blinding | Participant does not know their group assignment |
| 5 | Therapist blinding | Therapist does not know if they are delivering the experimental or control treatment |
| 6 | Assessor blinding | Pre and post testing is conducted by a person who does not know if participant received experimental treatment |
| 7 | Key outcome for 85% sample | Primary outcome measure is measured for at least 85% of participants |
| 8 | Treatment or control received as allocated | Explicit statement that participants received treatment or control condition as allocated |
| 9 | Between group comparisons | Between-group statistical comparisons are reported |
| 10 | Point to point and variability measures | Point measures and measure of variability (e.g., mean and standard deviation) are provided for at least one key variable |
| 11 | Treatment fidelity | Adherence to treatment protocol is assessed and reported |
| 12 | Treatment replicability | Treatment procedures are clearly described or made available |
| *Note*. NR = not rated. For further information see Cherney et al. (2013) and visit pedro.org.au | | |

**Table S3**

*Pedro+ Item Level Ratings Per Study*

| **Article** | **Eligibility (not rated)** | **Random group allocation** | **Allocation concealed** | **Groups similar baseline** | **Blinding of subjects** | **Blinding of therapists** | **Blinding of assessors** | **Outcome obtained >85%** | **Treatment received as allocated** | **Between-group comparisons** | **Point to point and variability measures** | **Treatment fidelity** | **Treatment replicability** | **PEDro+ total (max =12)** |
| --- | --- | --- | --- | --- | --- | --- | --- | --- | --- | --- | --- | --- | --- | --- |
| *Bo et al.., 2019 | 1 | 1 | 1 | 1 | 0 | 0 | 1 | 0 | 1 | 1 | 1 | 0 | 1 | 8 |
| Chan & Tsang, 2018 | 1 | 1 | 1 | 1 | 0 | 0 | 1 | 1 | 1 | 1 | 1 | 0 | 0 | 8 |
| *Colledge et al., 2017 | 0 | 0 | 0 | 1 | 0 | 0 | 0 | 0 | 1 | 1 | 1 | 0 | 0 | 4 |
| *El-Tamawy et al., 2012 | 1 | 0 | 0 | 1 | 0 | 0 | 0 | 1 | 1 | 1 | 1 | 0 | 1 | 6 |
| *Ezeugwu et al., 2018 | 1 | 0 | 0 | 0 | 0 | 0 | 0 | 1 | 1 | 0 | 1 | 1 | 1 | 5 |
| *Ihle-Hansen et al., 2019 | 1 | 1 | 0 | 1 | 0 | 0 | 0 | 1 | 1 | 1 | 1 | 0 | 1 | 7 |
| *Khattab et al., 2020 | 1 | 1 | 0 | 1 | 1 | 0 | 1 | 1 | 1 | 1 | 1 | 0 | 1 | 9 |
| *Kim & Yim, 2017 | 1 | 1 | 0 | 1 | 0 | 0 | 0 | 1 | 1 | 1 | 1 | 0 | 1 | 7 |
| *Kluding et al., 2011 | 1 | 0 | 0 | 0 | 0 | 0 | 0 | 0 | 1 | 0 | 1 | 0 | 1 | 3 |
| Krawcyk et al., 2019 | 1 | 1 | 1 | 1 | 0 | 0 | 1 | 1 | 0 | 1 | 1 | 1 | 0 | 8 |
| *Lee et al., 2018 | 1 | 0 | 0 | 0 | 0 | 0 | 0 | 1 | 1 | 1 | 1 | 0 | 0 | 4 |
| Macko et al., 2008 | 1 | 0 | 0 | 0 | 0 | 0 | 0 | 1 | 1 | 0 | 1 | 0 | 1 | 4 |
| *Marzolini et al., 2013 | 1 | 0 | 0 | 0 | 0 | 0 | 0 | 1 | 1 | 0 | 1 | 0 | 1 | 4 |
| *McDonnell et al., 2011 | 1 | 0 | 0 | 1 | 0 | 0 | 0 | 1 | 1 | 1 | 1 | 0 | 0 | 5 |
| Meester et al., 2019 | 1 | 1 | 0 | 1 | 0 | 0 | 1 | 1 | 0 | 1 | 1 | 0 | 0 | 6 |
| *Moore et al., 2015 | 1 | 1 | 0 | 1 | 1 | 0 | 1 | 1 | 1 | 1 | 1 | 0 | 1 | 9 |
| *Moriya et al., 2016 | 0 | 0 | 0 | 0 | 0 | 0 | 0 | 1 | 1 | 0 | 1 | 0 | 0 | 3 |
| Pallesen et al., 2019 | 1 | 1 | 1 | 0 | 1 | 0 | 1 | 0 | 1 | 1 | 0 | 1 | 1 | 8 |
| Ploughman et al., 2008 | 1 | 1 | 0 | 0 | 0 | 0 | 0 | 1 | 1 | 1 | 1 | 0 | 1 | 6 |
| Ploughman et al., 2019 | 1 | 1 | 1 | 1 | 1 | 0 | 1 | 1 | 1 | 1 | 1 | 0 | 1 | 10 |
| Quaney et al., 2009 | 1 | 1 | 0 | 1 | 0 | 0 | 1 | 1 | 1 | 1 | 1 | 0 | 0 | 7 |
| *Rosenfeldt et al., 2019 | 1 | 1 | 0 | 1 | 0 | 0 | 0 | 1 | 1 | 1 | 1 | 0 | 1 | 8 |
| *Swatridge et al., 2017 | 1 | 1 | 0 | 1 | 0 | 0 | 0 | 1 | 1 | 1 | 1 | 0 | 1 | 8 |
| Stuart et al., 2009 | 1 | 0 | 0 | 0 | 0 | 0 | 0 | 0 | 0 | 1 | 1 | 1 | 1 | 5 |
| Tang et al., 2016 | 1 | 1 | 0 | 1 | 1 | 0 | 1 | 0 | 1 | 1 | 1 | 0 | 0 | 8 |
| *Unibaso-Markaida et al., 2019 | 1 | 0 | 0 | 0 | 0 | 0 | 0 | 1 | 1 | 1 | 1 | 0 | 0 | 5 |
| *Yoo & Yoo, 2011 | 1 | 1 | 0 | 1 | 0 | 0 | 1 | 1 | 1 | 1 | 1 | 0 | 1 | 9 |
| # papers reporting item | 25 | 16 | 5 | 17 | 5 | 0 | 11 | 21 | 24 | 22 | 26 | 4 | 17 | Average = 6.39 |
| Note: * = studies that showed positive cognitive effects of aerobic exercise. 0 = high risk of bias/ not reported; 1 = low risk of bias/ reported | | | | | | | | | | | | | | |

| **Table S4**  *Description of Outcome Measures Found in Studies Reviewed* | | | |
| --- | --- | --- | --- |
| **Abbreviation** | **Test Name** | **Test Description** | **Study #** |
| SIS | Stroke Impact Scale | Self-report questionnaire that includes cognition and communication outcomes | 5, 9, 12, 16, 22, 23, |
| SS-QOL | Stroke Specific Quality of Life Scale | Self-report questionnaire that includes language and thinking domains | 27 |
| TMT A | Trail Making Test A | Visual attention | 6, 8, 14, 18, 19, 21, 26 |
| TMT B | Trail Making Test B | Visual attention and task switching | 1, 6, 7, 8, 14, 18, 19, 21, 25, 26 |
| SCWT | Stroop Color and Word Test | Inhibition | 1, 7, 8, 14, 21, 25 |
| MRT | Mental Rotation Test | Visuospatial skills | 1 |
| FDS | Forward Digit Span | Verbal short-term memory | 1, 14, 18, 25, 26 |
| BDS | Backward Digit Span | Verbal working memory | 7, 9, 14, 18, 25, 26 |
| ACE-R | Addenbrooke’s Cognitive Examination - Revised | Attention/orientation, memory, fluency, language and visuospatial skills | 4, 16 |
| MoCA | Montreal Cognitive Assessment | Short term memory, visuospatial skills, executive functions, attention, concentration, and working memory, language, and orientation to time and place | 5, 8, 10, 13, 15 |
| MMSE | Mini Mental State Exam | Orientation, attention, memory, language, and visuospatial skills | 6, 11 |
| FT | Flanker Task | Inhibition | 9, 24 |
| AST | Auditory Stroop Task | Inhibition | 2 |
| SVF | Semantic Verbal Fluency | Executive function, verbal production | 14 |
| SSpan | Spatial Span | Nonverbal short-term memory | 14 |
| RAVLT | Rey Auditory Verbal Learning Test | Verbal learning and memory | 3, 18 |
| CLOX | Clock drawing | Executive function, visuospatial skills, semantic memory | 14 |
| WCST | Wisconsin Card Sorting Task | Executive function | 21 |
| SRRT | Serial Reaction Time Task | Implicit learning | 21 |
| FCSRT | Free and Cued Selective Reminding | Episodic memory | 14 |
| PASAT | Paced Auditory Serial Addition Test | Attention | 14, 19 |
| IT | Inspection Time | Processing speed | 14 |
| mSWMT | Modified Sternberg Working Memory Task | Working memory | 17 |
| TOL | Tower of London | Executive function | 18 |
| SS | Serial Subtraction | Attention | 18 |
| DSST | Digit Symbol Substitution Test (a.k.a. Coding subtest from WAIS-IV) | Associative learning | 18, 19 |
| RCFT | Rey Complex Figure Test | Visuospatial ability and memory | 18 |
| RPMT | Raven's Progressive Matrices Test | Abstract reasoning | 20 |

*Note*. The cognitive and linguistic outcome measures used in each study were collated based on whether they were self-reported or quantitative/performance-based and on what domain of cognition they measured. Then effects on each measure from each study were tallied. See Figure 3 for results.
